# Chest compression quality decreases in hypoxic conditions simulating an airliner cabin at cruising altitude: a randomized, controlled, double-blind Manikin Study

**DOI:** 10.1038/s41598-024-77149-4

**Published:** 2024-10-29

**Authors:** Jan Schmitz, Daniel Aeschbach, Inga Beccard, Nina Frings, Jochen Hinkelbein, Jens Jordan, Tobias Kammerer, Felix Liebold, Ulrich Limper, Titiaan Post, Volker Schick, Jens Tank, Eva-Maria Elmenhorst

**Affiliations:** 1 Department of Anesthesiology and Intensive Care Medicine, University Hospital of Cologne, Kerpener Str. 62, 50937 Cologne, Germany; 2 Department of Sleep and Human Factors Research, Institute of Aerospace Medicine, German Aerospace Center, 51147 Cologne, Germany; 3 Department of Anaesthesiology, Intensive Care Medicine and Emergency Medicine, Johannes Wesling Klinikum Minden, University Hospital, Ruhr-University Bochum, Minden, Germany; 4 Institute of Aerospace Medicine, German Aerospace Center, 51147 Cologne, Germany; 5 Medical Faculty, University of Cologne, Cologne, Germany; 6 Department of Anesthesiology and Intensive Care Medicine, University Hospital of Leipzig, 04103 Leipzig, Germany; 7 Department of Anesthesiology and Intensive Care Medicine, Krankenhaus Merheim, Köln, Germany; 8 Department of Cardiovascular Medicine, Institute of Aerospace Medicine, German Aerospace Center, 51147 Cologne, Germany; 9 Institute for Occupational, Social, and Environmental Medicine, Medical Faculty, RWTH Aachen University, 52074 Aachen, Germany

**Keywords:** CPR, Hypoxia, Manikin, In-flight medical emergency, Resuscitation, Circulation, Preclinical research, Cardiovascular diseases

## Abstract

Air traveler numbers are predicted to reach 4.0 billion in 2024. Between 1/15,000–50,000 passengers will experience acute medical problems inflight with cardiac arrests requiring cardiopulmonary resuscitation (CPR) accounting for 0.3% of medical emergencies. Hypoxia in airplane cabins could impair oxygenation and physical performance of caregivers. We conducted a randomized controlled, double-blind study to test the hypothesis that hypoxia decreases the effectiveness in performing CPR. We randomized 24 healthcare professionals to two different study arms, each consisting of two conditions: arm (1) ‘hypoxia (FiO_2_ 15%, equivalent to 2400 m altitude)’ versus ‘normoxia’; arm (2) ‘hypoxia + supplemental oxygen’ versus ‘normoxia + supplemental oxygen’. The order of conditions was counterbalanced and a minimum wash-out period of 24 h was granted between conditions. In each condition participants performed a 5-min cardiac compression only CPR (CCO-CPR) using a full-body manikin after one, three and six hours in an altitude chamber. Mixed ANOVAs with post-hoc false-discovery-rate adjusted pairwise comparisons indicated that although compression frequency was maintained, the number of compressions with correct depth was decreased at all times during hypoxia compared to normoxia (all *p* < 0.002). After 6 h hypoxia exposure, mean compression depth was below the recommended compression depth defined by ERC/AHA guidelines and reduced compared to normoxia (42.4 ± 12.6 mm vs. 54.6 ± 4.3 mm, *p* < 0.0001). Supplemental oxygen during CCO-CPR in hypoxia prevented the decrease of compression-depth (55.3 ± 3 mm). Extended hypoxia exposure akin to conditions in airplane cabins can reduce quality of chest compressions during CPR. Supplemental oxygen for healthcare providers is an effective countermeasure.

## Introduction

The International Air Transport Association (IATA) expects overall traveler numbers to reach 4.0 billion in 2024, exceeding pre-COVID-19 levels^1^. With this, air traveler numbers have quadrupled since 2002^2^. Between 1 out of 15,000 to 50,000 passengers will experience acute medical problems/emergencies during a flight (i.e., in-flight medical emergency)^3^. The environmental conditions during a flight, mainly the decreased oxygen partial pressure in the aircraft cabin, but also the frequent consumption of alcohol inflight^4^, pose health risks for passengers especially if they have pre-existing medical conditions such as respiratory diseases^5^. Indeed, impaired lung function has been associated with the increased occurrence of sudden cardiac death^6^. Cardiac arrest accounts for approximately 0.3%, theoretically 800 events per year, of all in-flight medical emergencies and the risk of cardiopulmonary resuscitation (CPR) during air travel is increased dependent on flight duration^3^. Rapid recognition of cardiac arrest and early high-quality CPR is crucial for survival^7–9^.

Hypoxia decreases peripheral oxygen saturation and negatively affects physical performance^10–13^. CPR quality may be reduced in hypoxic environments. However, CPR quality seems also to depend on physical fitness^14^, as studies have shown, that the accumulated fatigue during a prolonged water rescue performed by lifeguards reduces the quality of chest compressions and ventilations on CPR^15,16^. It has been observed that highly trained individuals are able to maintain high quality CPR during the initial 10 min at altitudes up to 3100 m^17,18^. Therefore, supplemental oxygen and switching positions every 1 min to prevent rescuer fatigue have been suggested for CPR providers at altitudes above 1500 m^19^. However, few studies directly evaluated hypoxia-influences on CPR quality. Moreover, most studies examined alpine environments with oxygen fractions equivalent to > 3000 m. These studies showed that CPR performance was negatively affected at higher altitudes^20,21^. Furthermore, in a study simulating airplane cabin altitude by breathing a hypoxic gas mixture via facemask, CPR providers developed oxygen desaturations as low as 77%, fatigued more quickly, and performed CPR less well than in normoxia^22^. Finally, helicopter emergency medical services personnel operating in mountainous terrain have reported negative effects providing CPR at altitudes > 3000 m due to rapid ascents and hypoxia exposure^23^.

We therefore tested the hypothesis that exposure to hypoxia comparable to the conditions experienced during the flight in a commercial airliner decreases CPR quality of Emergency Medical Technician (EMT) personnel.

## Materials and methods

### Ethics

The study protocol was reviewed and approved by the ethics committee of the North Rhine medical board (Ärztekammer Nordrhein, Düsseldorf, Germany, number 2022313, date 01/2023) and conducted according to the principles of the Declaration of Helsinki and Good Clinical Practice. Written informed consent was obtained from all participants before enrollment. The study was registered on the German Clinical Trial Register on 07.05.2024 (DRKS00034159, https://drks.de/search/de/trial/DRKS00034159).

### Participants

Thirty male and female participants were invited to the lab for medical eligibility screening in December 2022. After completing a short questionnaire to gather information about general demographics (e.g. age, sex, height, weight), participants underwent medical examination. Participants were eligible when they held a board-certified EMT certificate (German: ‘Rettungssanitäter’), were within the age range of 18–45 years and had no acute or chronic health issue (especially with regard to a potential impact on CCO-CPR performance in a hypoxic environment). Exclusion criteria were any acute or chronic (ear-nose-throat-) diseases, any internal or surgical medical condition with impact on CPR performance, any permanent medication (except birth-control-pills), any acute infectious disease (including SARS-CoV-2, tested prior to entering the experimental site), any psychiatric condition which could preclude the participant from staying in an altitude chamber for at least 6 h (e.g., claustrophobia) and any possible pre-acclimatization to hypoxia as a stay at high altitude with a minimum of 6 weeks prior to the study start.

Twenty-four participants were selected and randomized into two arms by 1:1 ratio using an online randomizer software (Research Randomizer, https://www.randomizer.org/). Both groups did not differ regarding demographics (Table 1). Six participants were not eligible to participate (five because of acute medical conditions (flu, common cold) on the assigned study day, one because of permanent medication (thyroid hormone), which had not been stated before).

**Table 1:** Participants’ demographic parameters.

	Total	Hypoxia/normoxia group	Suppl. oxygen group	*p*-value
Sample size	24	12	12	NA
Female/male ratio	10:14	6:6	4:8	NA
Age [years]	25.04 ± 4	25.75 ± 4.48	24.33 ± 3.52	*p* = 0.3987
Height [m]	1.76 ± 0.09	1,74 ± 0.08	1.79 ± 0.1	*p* = 0.2137
Weight [kg]	75.54 ± 11.82	74.17 ± 12.73	76.92 ± 11.24	*p* = 0.5805
BMI [kg/m^2^]	24.26 ± 2.8	24.42 ± 06	24.09 ± 2.63	*p* = 0.7763

*NA* not applicable, *BMI* body mass index.

Mean ± standard deviation; *p* < 0.05 was considered significant in an independent t-test.

### Protocol

**Fig. 1:**
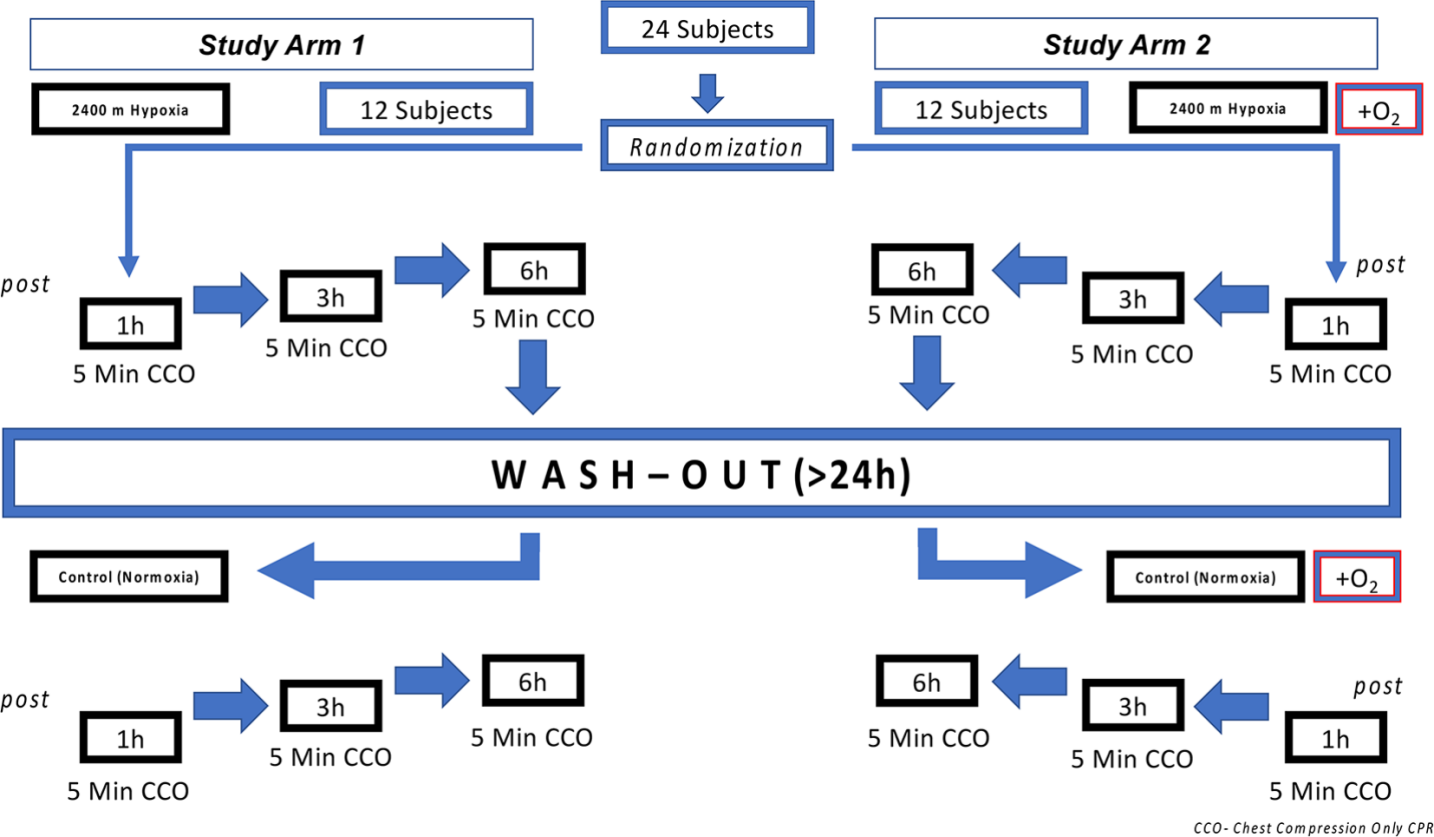
Flowchart of study protocol and randomisation process.

We conducted the study in the altitude chamber at the ‘:envihab’ of the German Aerospace Center (DLR) (https://www.dlr.de/envihab/), Cologne, Germany. We report our results in concordance to the CONSORT reporting guidelines^24^. The protocol is presented in Fig. 1.

After physical examination and giving written informed consent, three participants at a time entered the altitude chamber.

In arm 1, the altitude chamber contained a fraction of inspired oxygen (FiO_2_) of 15% in the hypoxia condition or a FiO_2_ of 20.9% in the normoxia condition. Each of the 12 participants underwent both conditions in a counterbalanced order and with a wash-out period of at least 24 h between conditions.

In arm 2, the identical atmospheric conditions were presented as in arm 1, but participants wore face masks (covering mouth and nose) with oxygen reservoir (Dahlhausen^©^, Cologne, Germany) only during each 5-min CPR period and received supplemental oxygen at a flow rate of 6 l/min resulting in an estimated FiO_2_ of 40%; i.e. hypoxia + supplemental oxygen and normoxia + supplemental oxygen. Twelve additional participants underwent these two conditions in a counterbalanced order and with a wash-out period of at least 24 h between conditions. During each of the four conditions participants stayed in the altitude chamber for 6 h and 10 min in total. After 60 min of exposition, participants performed a continuous 5-min set of CCO-CPR. Subsequently, they rested for the upcoming sets of 5-min CCO-CPR which were scheduled after 180 min and 360 min.

We achieved hypoxia by nitrogen dilution through the air conditioning system in the atmospheric self-sustaining altitude chamber under normobaric conditions (1013 hPa). Nitrogen was supplied by an external tank. We exposed participants to normobaric hypoxia without pre-acclimatization (see exclusion criteria), thus simulating the rapid ascent of a commercial airplane to cruising altitude. The oxygen fraction of 15% corresponds to the oxygen partial pressure present in a pressurized aircraft cabin and is equivalent to an altitude of approximately 2400 m. Other environmental factors (i.e., noise, lighting) were maintained the same in all conditions. Atmospheric conditions (normoxia versus hypoxia) were not distinguishable for participants nor staff inside the altitude chamber due to equal noise levels of the air conditioning system in both conditions. So, both participants and staff inside the altitude chamber were blinded.

The altitude chamber was operated by one external technician.

### Data collection

Chest compressions were performed using a full-body manikin (AmbuMan^®^ Basic iQF, Ambu Ltd., Bad Nauheim, Germany) on the ground with sufficient light. Participants performed the chest compressions in an area of about 4 m^2^, thus, space was not as limited as in an airliner cabin. The iQF application (Ambu^®^, Ambu Ltd., Bad Nauheim, Germany) was run on an iPad Pro 2020 (Apple Inc., Cupertino, California, USA) monitoring data-driven information and accurate measurements of relevant CPR parameters as well as storage of individual CPR data. The application provided 5-min average values of CPR parameters. Also, vital parameters (heart rate and SpO_2_) were measured by fingertip sensor and one-lead electrocardiography and was recorded via a wireless monitor (Philips MX40, Philips Medical Systems, Hamburg, Germany) every 30 s in an Excel-file for each participant.

### Data availability

All data generated or analyzed during this study are included in this published article.

### Statistics

A statistical power analysis (GPower 3.1 software) was performed a priori for sample size estimation. Assuming a mean difference of 3% in compression depth with a standard deviation of 3.3% (Cohens d = 0.9) [at t = 6 h], *n* = 12 individuals needed to be analyzed to achieve 80% power based on a paired t-test (two-sided) and alpha-level of 5%. To minimize pre-existing differences in chest compression quality quality due to experience and training, we only included participants with board certified medical training (EMT personnel and emergency physicians).

Data was processed with Excel for Mac 16.32 (Microsoft^©^, Redmond, USA) and statistical analyses were performed with SAS (version 9.4).

The demographic parameters sex, weight, height, and age were compared between groups using independent t-tests.

The following parameters were used to assess chest compression quality.

The primary endpoint was:

- mean compression depth (mm) at timepoint 6 h of exposure.

The secondary endpoints were:

- mean compression depth (mm) at timepoints 1 h and 3 h of exposure.
- mean compression frequency (number/min).
- number of compressions with correct compression depth.
- number of compressions with too deep compression depth (> 60 mm).
- number of compressions with too low compression depth (< 50 mm).
- number of missing chest releases (> 20 mm depth before start of next compression).
- Insufficient-flow-time (> 2 s with no chest compression).
- peripheral O_2_-saturation, SpO_2_ (%), mean SpO_2_ averaged over 5 min at each timepoint as well as 30-s averages at the beginning, in the middle, and at the end of the 5-min CCO-CPR.
- heart rate (beats per minute), mean heart rate averaged over 5 min at each timepoint as well as 30-s averages at the beginning, in the middle, and at the end of the 5-min CCO-CPR.

We used random subject mixed ANOVAs with the factors “condition” (hypoxia, normoxia, hypoxia + supplemental oxygen, normoxia + supplemental oxygen), “timepoint” (1 h, 3 h, and 6 h) and the interaction between “condition x timepoint” for analyses of endpoints. Post-hoc pairwise comparisons (in total 24 planned comparisons of interest: three within conditions hypoxia, normoxia, and hypoxia + supplemental O_2_ (3 × 3 = 9), and three between conditions at each timepoint (3 × 5 = 15)) were adjusted for multiple comparisons with the False-Discovery-Rate. Transformations were used to achieve normal distribution of residuals. Compression depth and compression frequency were squared, number of too low compressions was square root transformed, number of too deep compressions, number of missing chest releases and mean heart rate (averages over 5 min at each timepoint) were log-transformed, mean SpO_2_ (averages over 5 min at each timepoint) were third potency transformed. In case of significant effects for mean heart rate or mean SpO_2_, data were further compared at the beginning, the middle and the end of each timepoint with Wilcoxon signed rank tests for paired comparisons or Wilcoxon rank-sum tests for unpaired comparisons.

Results were considered significant if *p* < 0.05. All findings are presented as means ± standard deviation (p-value) if not stated otherwise.

## Results

### Participants

Demographic parameters did not differ between groups (Table 1).

### Vital parameters

#### Supplemental oxygen prevents the decrease of SpO2 observed in hypoxia

Mixed ANOVA of mean SpO_2_ indicated differences between conditions (*p* < 0.0001), but not between timepoints (*p* = 0.7078) or for the interaction (*p* = 0.1092). Mean SpO_2_ was decreased at all timepoints in hypoxia (1 h: 89.7%±2.5%; 3 h: 89.0%±3.7%; 6 h: 91.0%±5.1%) compared to normoxia (1 h: 96.4%±2.7%; 3 h: 96.1%±3.2%; 6 h: 94.3%±5.5%) (all *p* < 0.005). SpO_2_ was lower in hypoxia vs. normoxia in all 30-s averages at the beginning, the middle and the end of each timepoint (all *p* < 0.05) except for the middle of hour six where SpO_2_ was on trend-niveau decreased (*p* = 0.0874).

Supplemental oxygen in hypoxia increased mean SpO_2_ (1 h: 96.1%±4.2%; 3 h: 96.3%±5.1%; 6 h: 97.4%±2.8%) compared to hypoxia at all timepoints (all *p* < 0.0015). SpO_2_ in the hypoxia + supplemental oxygen condition was higher compared to the hypoxia condition at the beginning, the middle and the end of each timepoint (all *p* < 0.025).

Mean SpO_2_ did not differ between the normoxia compared to the hypoxia + supplemental oxygen condition, as well as for the normoxia compared to normoxia + supplemental oxygen condition (1 h: 96.1%±4.3%; 3 h: 96.6%±4.7%; 6 h: 96.9%±3.4%) at all timepoints (all *p* > 0.12).

#### Hypoxia did not increase heart rate

Mixed ANOVA of mean heart rate indicated differences between conditions (*p* = 0.0005) and timepoints (*p* = 0.0167) but not for the interaction (*p* = 0.9854). However, after adjustment for multiple testing no significant pairwise comparisons were detected (all *p* > 0.2).

### Chest compression quality

**Fig. 2:**
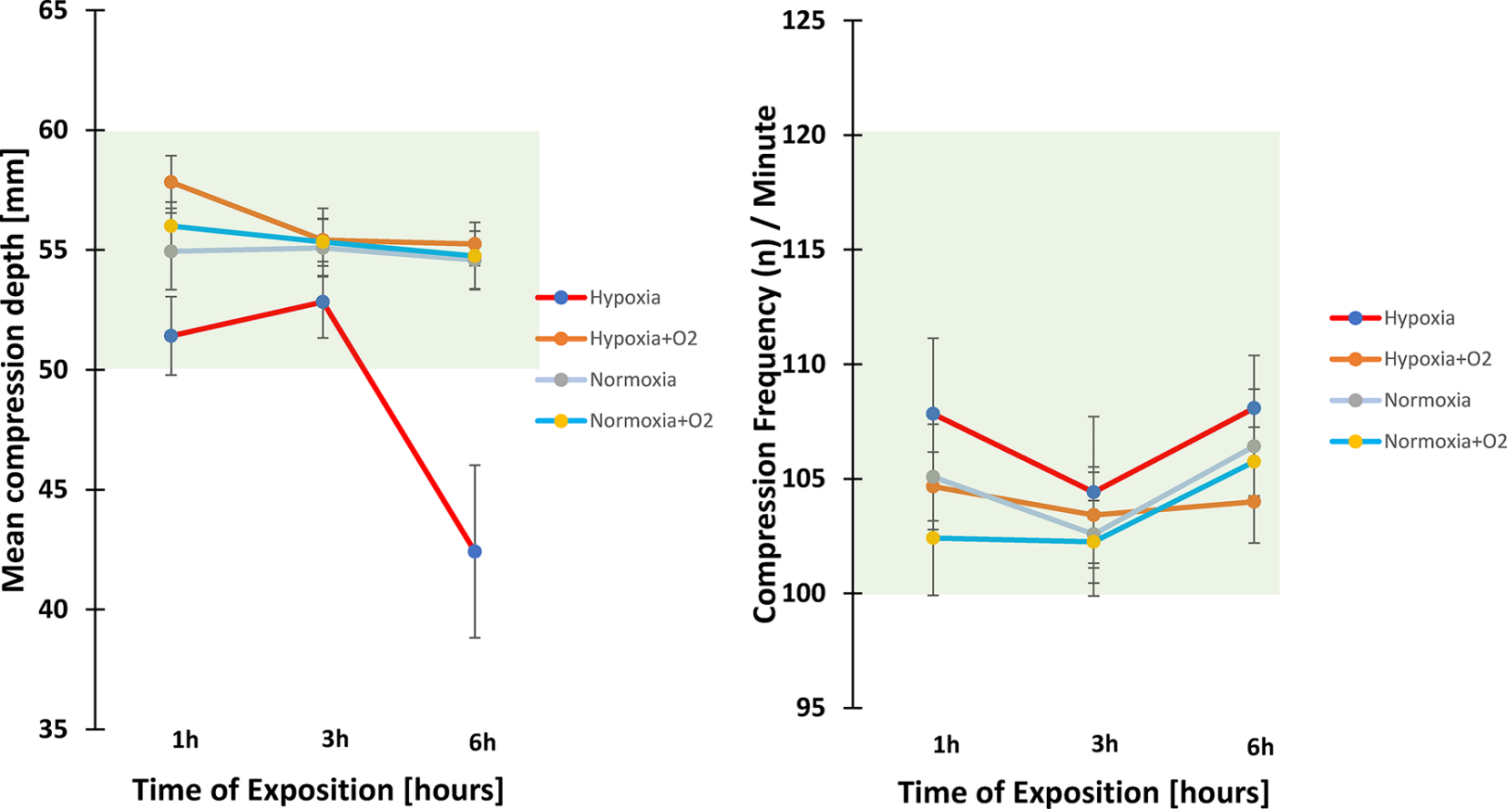
Mean compression depth and frequency. Left: Comparison of mean compression depth after 1-h, 3-h, and 6-h exposure to the four study conditions. Right: Comparison of mean compression frequency after 1-h, 3-h, and 6-h exposure to the four study conditions. The green boxes represent the recommended compression depth and frequency according to ERC/AHA guidelines. mean + standard deviation.

Figure 2 displays mean compression depth and frequency in all conditions and time points.

#### Hypoxia decreases compression depth: Hypoxia vs. Normoxia

Mean compression depth in normoxia was within the recommended CPR guideline range of 50–60 mm at all timepoints (Table 2).

**Table 2:** Primary and secondary endpoints evaluating CPR quality in four different conditions.

	Hypoxia			Normoxia			Hypoxia + O2			Normoxia + O2			ANOVA		
	1 h	3 h	6 h	1 h	3 h	6 h	1 h	3 h	6 h	1 h	3 h	6 h	Condition	Timepoint	Interaction
Compression depth [mm]	51.4 ± 5.7	52.8 ± 5.2	42.4 ± 12.6	54.9 ± 5.7^b^	55.1 ± 4.3	54.6 ± 4.3^b^	57.8 ± 3.8^a^	55.4 ± 3.2	55.3 ± 3^a^	56 ± 3.6	55.3 ± 4.7	54.8 ± 4.8’	**< 0.0001**	**< 0.0001**	**0.0003**
Compression frequency [number/min]	107.8 ± 11.5	104.4 ± 11.3	108.1 ± 7.9	105.1 ± 8	102.6 ± 9.5	106.4 ± 8.5	104.7 ± 5.1	103.4 ± 7.3	104 ± 6.2	102.4 ± 8.6	102.3 ± 6.3	105.8 ± 5.3	0.3853	0.1305	0.9339
Compressions with correct compression depth [number]	264.3 ± 164.6	352.3 ± 116.3	165.8 ± 168.6	445.7 ± 110^b^	445.5 ± 77^b^	460 ± 76.9^b^	450.8 ± 96.9	462.3 ± 52.9	489.2 ± 34.4	471.4 ± 46’	457.7 ± 59.7’	461.7 ± 56.5’	**> 0.0001**	0.6648	**0.0364**
Compressions with too deep compression depth [number]	23.8 ± 63.9	21.7 ± 70.4	12.7 ± 39.9	16.3 ± 30.7	22.1 ± 63.5	8.4 ± 18.2	45.5 ± 98.2	20.3 ± 57.2	2.4 ± 3.9	18.3 ± 38.2	17.3 ± 43.9	26.3 ± 43.2	0.4350	0.2555	0.9327
Compressions with too low compression depth [number]	228.1 ± 190.9	148.1 ± 133.4	353.2 ± 200.2	63.3 ± 96.3^b^	46.3 ± 61.8^b^	63.8 ± 68^b^	21.7 ± 31.3^a^	34.4 ± 38.8^a^	28.4 ± 35^a^	23.1 ± 38.9’	35.5 ± 63.1’	41.1 ± 68.6’	**< 0.0001**	**0.0044**	**0.0143**
Compressions with missing chest releases [number]	3.5 ± 8.3	9 ± 14.7	9 ± 18	14.3 ± 19.3	10.3 ± 15.4	17.7 ± 24.9	13.2 ± 22.7	14.3 ± 21.5	14.5 ± 23.25	16.1 ± 28.1	8.3 ± 12.9	13.6 ± 21.9	0.1109	0.9692	0.7528
Periods of no-flow-time [number]	–	–	–	–	–	–	–	–	–	–	–	–			

This table provides an overview of descriptive and ANOVA statistics of CPR quality. Mixed ANOVAs of mean compression frequency, number of compressions with too deep compression depth and missing releases did not indicate differences between conditions, timepoints, and their interaction. CPR frequency was in concordance with the current CPR-guidelines. Intervals without cardiac compression longer than 2 s (No-flow-time) were not observed. Mean ± standard deviation.

Hypoxia vs. Normoxia + O2 at respective timepoints.

Significant values are in bold.

^a^Hypoxia + O2 vs. Hypoxia at respective timepoints.

^b^Normoxia vs. Hypoxia at respective timepoints.

In hypoxia mean compression depth was decreased at timepoints 1 h (*p* = 0.0357) and 6 h (*p* = 0.0005), corresponding to a mean difference of 22.7%±19.9% after 6 h in hypoxia compared to normoxia. The number of compressions with correct compression depth was reduced and the number of compressions with too low compression depth was increased at all timepoints (all *p* < 0.002) compared to normoxia.

Mean compression depth was decreased and number of compressions with too low compression depth was increased after six hours in hypoxia compared to one and three hours in hypoxia (all *p* < 0.003) and fell below the recommended range at that timepoint. Number of compressions with correct pressure depth was reduced after 6 h compared to 3 h in hypoxia (*p* = 0.0021).

#### Supplemental oxygen successfully counteracted the hypoxia-induced CPR-quality decrease: Hypoxia vs. hypoxia + O2

Mean compression depth in the hypoxia + supplemental oxygen condition was improved after 1 h (*p* = 0.0144) and 6 h (*p* = 0.0005) compared to the hypoxia condition and stayed in the recommended range. Number of compressions with correct compression depth was increased and number of compressions with too low compression depth was reduced at all timepoints in hypoxia + supplemental oxygen vs. hypoxia (all *p* < 0.025). Thus, administered supplemental oxygen was able to reverse the hypoxia-induced impairment in CPR quality.

#### Supplemental oxygen in hypoxia restores performance to normoxic level: Normoxia vs. hypoxia + O2

Mean compression depth, number of compressions with correct compression depth, and number of compressions with too low compression depth did not differ in hypoxia + supplemental oxygen vs. normoxia at all timepoints (all *p* > 0.3).

#### Hyperoxia does not improve normoxic CPR performance: Normoxia vs. normoxia + O2

Mean compression depth, number of compressions with correct compression depth and number of compressions with too low compression depth did not differ in normoxia + supplemental oxygen vs. normoxia at all timepoints (all *p* > 0.3). Thus, supplemental oxygen in normoxia was neither able to improve nor alter chest compression quality during CPR. Chest compression quality was within the AHA/ERC recommendations in both conditions.

## Discussion

The important finding of our study is that hypoxia exposure resembling conditions in an airplane cabin decreased the number of correctly executed CPR compressions by experienced medical professionals after 1 h, 3 h, and 6 h compared with normoxic conditions. Hypoxia did not affect chest compression frequency, but the depth of compressions such that the number of compressions which was determined as being too low was increased at all timepoints. Indeed, mean chest compression depth failed to fulfil current CPR guidelines after 6 h which was not the case after 1 h and 3 h in hypoxia. This is a clear indication of a time-dependent effect of hypoxia on chest compression performance during CPR. Hypoxia exposure led to a physiologically relevant SpO_2_ decrease to between 89 and 91% during CPR, levels that can be expected to negatively impact physical performance^25^. Values have oscillated just around the 90% SpO_2_ threshold used in clinical guidelines to mark the state of hypoxia^26^. Supplemental oxygen was an effective countermeasure against the hypoxia-induced SpO_2_ decrease and performance loss. Both were maintained at levels observed under normoxia conditions.

We conducted our study in randomized and double-blind fashion to attenuate potential biases among study participants and investigators on study outcomes. Moreover, we applied ambient normobaric hypoxia which provides a more realistic CPR setting compared to applying hypoxia via face masks^22^. With the normoxia + supplemental oxygen condition as comparator, however, we showed that wearing a face mask per se did not negatively impact CPR quality. This is important as supplemental oxygen during CPR in the airliner cabin would most likely be provided via face mask. We also showed that supplemental oxygen was only able to improve CPR quality in a hypoxic state but not in a normoxic environment.

Previous studies primarily conducted for alpine environment showed a decrease in CPR quality when performing CPR at higher altitudes, especially when conducted in an altitude of more than 3000 m^20,21^. Another study^23^, which had a similar methodological approach also using 5 min CCO-CPR, showed a decrease in chest compression depth after 20 min depending on different simulated altitudes (3000 m and 5000 m vs. 200 m), but no significant difference in chest compression frequency. Exhaustion due to hypoxia exposure appears to primarily affect compression depth rather than compression frequency, which was attributed to exhaustion when performing CPR, regardless of environmental conditions^27,28^. Our results, however, indicate that the hypoxia-induced decrease of SpO_2_ in a hypoxic environment is an important factor mediating CPR quality decrease. For professional healthcare providers it seems easier and more reliable to focus on maintaining the correct CPR frequency than the correct depth. This phenomenon is well known from several other studies^21,29^ in a normoxic environment and also described in the present AHA/ERC-guidelines^7,30^. Even metronome guided chest compression rates did not improve chest compression quality that was impaired by rescuer fatigue^31^. Yet, physical exhaustion not caused by hypoxia but by water rescue showed, that exhaustion can also paradoxically increase CPR frequency^15^. This study compared the quality of 5-min CPR under rested and exhausted conditions, and showed that CPR frequency was higher for exhausted providers (exhausted, 411 ± 56.09 compressions per 5 min; rested, 380 ± 38.64 compressions per 5 min; *p* < 0.001). The higher CPR frequency was probably a consequence of the subjectively perceived lower CPR compression depths^15^.

Physical fitness appears to have some influence on CPR performance, as Neset and colleagues have shown by testing CPR quality of elderly laypersons, aged 50–75^32^. However, elderly laypersons, with a reduced physical fitness compared to younger individuals, were still capable of performing chest compressions with acceptable quality for 10 min in a realistic cardiac arrest simulation^32,33^. In our study, CPR performance of young and healthy healthcare providers was reduced under hypoxia, emphasizing the importance of taking the environmental conditions into account under which CPR is to be delivered.

To maintain high-quality CPR during hypoxia, one likely approach would be to supply supplemental oxygen via face mask. Another possibility would be to shorten CPR cycles, if additional providers are on board. Shortening CPR cycles to less than 2 min per provider has been shown to improve CPR quality in case of fatigue during prolonged CPR duration^34^. Obviously, this effect is only present in situations, when CPR providers are negatively affected by exhaustion^35^.

## Limitations

An important limitation of our study is that CPR was conducted under laboratory conditions with manikins, which may not produce the psychological stress and neurohumoral activation of real CPRs on commercial airline flight^36,37^. Moreover, we did not simulate the limited space inside an aircraft cabin, which might influence CPR performance. Furthermore, our study was conducted with health care professionals experienced in CPR, which cannot be easily translated to less experienced persons conducting CPR inflight. Our results should be verified in further studies in a more realistic simulated cabin environment. Our results are limited to the three timepoints that we scheduled for CPR. It remains unclear, when after the beginning of hypoxia exposure CPR compression depth would drop below the current recommended guidelines, and whether CPR quality would continue to worsen during longer flight durations (more than 6 h). We suggest to examine these questions in specifically designed future studies. Also, a regular BLS-scenario containing chest compression and ventilation would be a more realistic scenario. Since this study was focusing on the effect of hypoxia on basic CPR parameters (inter alia frequency and depth) we decided to focus on one single part of BLS to rule out possible other effects which may alter CPR quality. We suggest to examine these questions in specifically designed future studies.

## Conclusion

Extended hypoxia exposure akin to conditions in commercial airliner cabins reduced the quality of CPR performed by experienced healthcare professionals. Supplemental oxygen had a beneficial effect on CPR quality. Our findings suggest that CPR guidelines for providers with an extended exposure to hypoxia, such as long-haul air travel or stay at higher altitudes, should be scrutinized and adjusted. Supplemental oxygen administration for CPR providers, shorter chest compression cycles, and mechanical CPR devices could be effective countermeasures against physical exhaustion. However, additional training requirements and costs of mechanical CPR devices have to be weighed against the relatively low incidence of inflight cardiac arrests. Oxygen supplementation may be a more realistic approach to maintain CPR performance during air travel.

## Data Availability

All data generated or analyzed during this study are included in this published article.

## Abbreviations

BMI: Body mass index
bpm: Beats per minute
CCO: Cardiac compression only
cm: Centimeter
CPR: Cardiopulmonary resuscitation
EMT: Emergency medical technician
FiO_2_: Fraction of inspired oxygen
IATA: International Air Transport Association
mm: Millimeter
s: Second
SpO2: Peripheral capillary oxygen saturation
